# Facteurs prédictifs de l’échec du Traitement Préventif Intermittent du paludisme à la sulfadoxine – pyriméthamine (TPIp-SP) dans une population de femmes enceintes à Yaoundé

**DOI:** 10.11604/pamj.2016.23.152.7936

**Published:** 2016-03-31

**Authors:** Félix Essiben, Pascal Foumane, Marcelle Aurelie Tsafack de Nguefack, Filbert Eko Eko, Philip Nana Njotang, Robinson Mbu Enow, Emile Telesphore Mboudou

**Affiliations:** 1 Département de Gynécologie-Obstétrique, Faculté de Médecine et des Sciences Biomédicales de l'Université de Yaoundé, Cameroun; 2 Hôpital Central de Yaoundé (HCY), Cameroun; 3 Hôpital Gynéco-obstétrique et Pédiatrique de Yaoundé, Cameroun; 4 Institut de Technologie Médicale de Yaoundé; 5 Hôpital Gynéco-Obstétrique et Pédiatrique de Douala, Cameroun

**Keywords:** Facteurs de risque, paludisme, traitement préventif intermittent, sulfadoxine-pyriméthamine, Cameroun, Risk factors, malaria, intermittent preventive treatment, sulphadoxine-pyrimethamine, Cameroon

## Abstract

**Introduction:**

Le traitement préventif intermittent à la sulfadoxine-pyriméthamine (TPIp-SP) est recommandé pour prévenir le paludisme pendant la grossesse. Nous avons recherché les facteurs associés à l’échec de cette stratégie.

**Méthodes:**

Nous avons mené une étude cas - témoins dans deux formations sanitaires de Yaoundé, du 1er Mai 2014 au 30 Avril 2015. Les femmes enceintes sous TPIp-SP hospitalisées pour paludisme ayant un Test de Diagnostic Rapide (TDR) positif (cas) étaient comparées aux femmes enceintes sous TPIp-SP ayant un TDR négatif (témoins). Les logiciels Epi info 7 et SPSS 18.0 ont été utilisés avec P < 0,05 comme seuil de significativité.

**Résultats:**

Nous avons recruté 234 sujets, 109 cas (46,6%) et 125 témoins (53,4%). Les facteurs associés retrouvés étaient: la primiparité (P=0,03; OR=1,15; IC= 0,32 - 4,10), la non utilisation de la MILDA (P=0,006; OR= 2,31; IC= 1,26 - 4,25), un antécédent d'hospitalisation pour paludisme (P=0,007; OR= 2,19; IC= 1,23 - 3,89), le début de la TPIp-SP après la 28ème semaine de grossesse (P=0,001, OR= 3,55; IC= 1,7 - 7,61). Après régression logistique, la primiparité (P=0,024; OR=2,01; IC=1,1-3,7) et un antécédent d'hospitalisation pour paludisme (P=0,001; OR=2,83; IC=1,50-5,4) restaient associés à l’échec du TPIp-SP.

**Conclusion:**

Un antécédent d'hospitalisation pour accès palustre et la primiparité sont des facteurs prédictifs indépendants de l’échec de la TPIp-SP.

## Abstract (en)

**Introduction:**

Intermittent preventive treatment with sulfadoxine-pyrimethamine (IPTp-SP) is recommended for malaria prevention during pregnancy. We investigated factors associated with the failure of this strategy.

**Methods:**

We conducted a case-control study in two health care facilities in Yaounde, from 1 May 2014 to 30 April 2015. Pregnant women treated with IPTp-SP, hospitalized for malaria and having a positive Rapid Diagnostic Test (RDT) result (case-subjects) were compared to pregnant women treated with IPTp-SP having a negative RDT result (control-subjects). Epi Info 7 software and SPSS 18.0 software were used with P< 0.05 as significance threshold.

**Results:**

We recruited 234 subjects, 109 (46.6%) case-subjects and 125 control-subjects (53.4%). The associated factors found were: prematurity (P=0.03; OR=1.15; IC= 0.32 – 4.10), the non-use of LLIN (P=0.006; OR= 2.31; IC= 1.26 – 4.25), a history of hospitalization for malaria (P=0.007; OR= 2.19; IC= 1.23 – 3.89), IPTp-SP administration after 28 gestational weeks (P=0.001, OR= 3.55; IC= 1.7 – 7.61). After logistic regression, prematurity (P=0.024; OR=2.01; IC=1.1-3.7) and a history of hospitalization for malaria (P=0.001; OR=2.83; IC=1.50-5.4) remained associated with IPTp-SP failure.

**Conclusion:**

A history of hospitalization for malaria and prematurity are independent predictor of IPTp-SP failure.

## Introduction

A travers l'Afrique et particulièrement au Cameroun, le paludisme en grossesse est un problème de santé publique majeure parce qu'il sévit de manière endémique et qu'il est responsable de complications graves pour la mère et l'enfant [1]. La femme enceinte et l'enfant sont particulièrement exposés. Les modifications physiologiques et immunitaires de la grossesse rendent les femmes enceintes vulnérables face à cette maladie [2]. L'utilisation de la Moustiquaire Imprégnée à Longue Durée d'Action (MILDA), le Traitement Préventif Intermittent pour le paludisme à la Sulfadoxine-Pyriméthamine (TPIp-SP) et une prise en charge adéquate des cas grâce à un traitement rapide du paludisme chez la femme enceinte sont des stratégies de prise en charge adoptées pour la prévention du paludisme en grossesse [3]. Le TPIp-SP est aussi bénéfique qu'efficace dans la prévention du paludisme en grossesse [4–7]. LOMS recommande que toutes les femmes enceintes doivent recevoir la SP à chaque consultation prénatale [3]. La chimioprophylaxie avec la SP est, avec la MILDA, la mesure de prévention la plus utilisée au Cameroun [6–8]. Son administration optimale pourrait diminuer le taux d’échec de cette prévention. Le TPIp-SP est le seul des trois volets relatifs à la prévention des complications du Paludisme en grossesse qui peut être directement influencé par le prestataire de soins. Parce que plusieurs contraintes peuvent influencer le respect de ces recommandations et compromettre l'efficacité de ces approches [9], l'accès palustre en grossesse continue d’être une cause fréquente d'hospitalisation dans notre milieu. Le but de cette étude est de rechercher les facteurs cliniques associés à la survenue de l'accès palustre chez les femmes enceintes sous TPI.

## Méthodes

**Type d’étude et cadre de l’étude:** nous avons mené une étude cas-témoins dans les services de Maternité de l'hôpital de District de Biyem-Assi et du Centre Hospitalier Catholique Nicolas Barré d'Ekounou, deux formations sanitaires de la ville de Yaoundé, du 1er Mai 2014 au 30 Mai 2015. **Population d’étude:** le groupe des cas était constitué des femmes enceintes sous TPIp-SP hospitalisées et traitées pour accès palustre, et ayant un TDR positif. L'accès palustre a été considéré comme étant l'apparition de signes cliniques du paludisme. Le groupe des témoins était constitué de femmes enceintes sous TPIp-SP ayant un TDR négatif. Le TDR a une spécificité pouvant atteindre 100% [10]. Les cas étaient recrutés consécutivement au moment de leur hospitalisation et le même jour les témoins étaient sollicités en CPN. Les femmes ayant déjà eu un épisode d'accès palustre pendant la grossesse en cours ou ayant un âge gestationnel inférieur à 16 semaines d'aménorrhée n’étaient pas incluses dans l’étude. **Collecte des données et paramètres étudiés:** le résultat du TDR a permis de repartir les femmes en fonction des deux groupes: le groupe des cas lorsque le test était positif en présence des signes d'accès palustre et le groupe des témoins lorsqu'il était négatif en absence des signes du paludisme. Par la suite, les données sociodémographiques et cliniques des femmes ont été collectées sur une fiche individuelle anonyme pré établie. Les variables d'intérêt étudiés ont été l’âge maternel, la parité, le statut HIV, la non utilisation de la MILDA, les antécédents d'hospitalisation pour accès palustre, l’âge de la grossesse au début du TPI et le nombre de doses de SP déjà prises. **Analyses statistiques:** afin d'identifier les facteurs prédictifs à l’échec du TPI, les patientes ayant fait un accès palustre sous TPI étaient comparées aux femmes enceintes régulièrement sous TPI. Les données ont été analysées à l'aide des logiciels Epi info7, SPSS 18.0 et Microsoft excel 2010. Les résultats ont été présentés sous forme de moyennes (± écart-type) pour les variables quantitatives et de fréquences pour les variables qualitatives. La comparaison des variables à l'aide du test de Fischer et du Chi carré de Pearson. La différence était statistiquement significative pour P < 0,05. Le rapport des côtes (Odd Ratio-OR) et son intervalle de confiance à 95% ont permis d’évaluer l'association entre les variables et la survenue de l'accès palustre. **Considérations éthiques:** la participation à l’étude était volontaire. Le consentement était libre et éclairé, écrit ou verbal. La clairance éthique a été obtenue du comité d’éthique institutionnel de l'Université de Douala de même que les autorisations administratives des deux formations sanitaires. Le TDR a été réalisé gratuitement et les femmes ont été informées du résultat obtenu.

## Résultats

**Figure 1:**
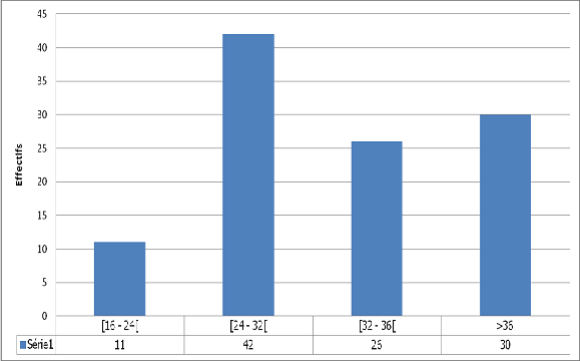
Distribution des femmes enceintes en fonction de l’âge gestationnel au moment de l'accès palustre (n=109)

Pendant une période de 12 mois, nous avons recruté 234 femmes enceintes, soit 109 cas (46,58%) et 125 témoins (53,42%). La moyenne d’âge était semblable dans les deux groupes (t=0,99; p > 0,9). L'accès palustre est apparu chez 54,1% des cas au delà de 45 jours après la dernière prise de SP et chez 72,2% des femmes lorsque ce délai était supérieur à 60 jours (Figure 1). Les facteurs de risque de l’échec du TPI identifiés (Tableau 1) étaient: La parité (P=0,03; OR=1,15; IC= 0,32 - 4,1), la non utilisation de la MILDA (P=0,006; OR= 2,31; IC= 1,26 - 4,25), un antécédent d'hospitalisation pour paludisme (P=0,007; OR= 2,19; IC= 1,23 - 3,89), la prise de la 1^ère^ dose de SP après la 28 ^ème^ semaine d'aménorrhée (P=0,001, OR= 3,55; IC= 1,7 - 7,61); la prise de moins de trois doses de SP (P=0,004; OR=2,78; IC=1,35 - 5,72). L'antécédent d'hospitalisation pour accès palustre et la parité sont des facteurs associés à l’échec de la TPIp-SP indépendamment du nombre de doses de SP prises, de la non utilisation de la MILDA et de l’âge gestationnel au début du TPIp-SP (Tableau 2).

**Tableau 1:** Comparaison des variables entre les femmes atteintes d'accès palustre sous TPI (groupe des cas ; n=109) et les femmes sous TPI sans accès palustre (groupe des témoins ; n=125)

Variables	Cas n (%)	Témoins n (%)	*P* value	Odds ratio (* IC 95%)
**Age maternel**				
<20	10 (9,2)	7 (5,6)	0,294	1,7(0,62-4,63)
[20 - 30[	73 (67,0)	76 (60,8)	0,327	1,31(0,77-2,27)
[30 - 40[	22 (20,2)	40 (32,0)	0,041	**0,54(0,3-0,98)**
〉 40	4 (3,7)	2 (1,6)	0,317	2,34(0,42-13,03)
**Parité**				
<1	45 (41,3))	34 (27,2)	0,023	**1,88(1,09-3,25)**
≥ 1	64 (58,7)	91 (72,8)		**0,53(0,31-0,92)**
**Age gestationnel**				
< 20 SA	2 (1,8)	3 (2,4)	0,764	0,76(0,12-4,63)
20 – 28 SA	33 (30,3)	29 (23,2)	0,221	1,44(0,8-2,58)
28 – 37 SA	49 (45,0)	64 (51,2)	0,340	0,78(0,47-1,31)
〉 37 SA	25 (22,9)	29 (23,2)	1,000	0,99(0,54-1,82)
**Statut matrimonial**				
Célibataire	52(47,7)	68(54,4)	0,308	0,76(0,45 – 1,27)
Noncélibataire	57(52,3)	57(45,6)		1,31(0,78 – 2,19)
**Statut VIH**				
Positif	5 (4,6)	5 (4,0)	0,823	1,15(0,32-4,08)
Négatif	104 (95,4)	120(96,0)		0,87(0,25-3,09)
**MILDA**				
Non	36 (33,0)	22 (17,6)	0,006	2,31(1,26 – 4,25)
Oui	73 (67,0)	103 (82,4)		0,43(0,23-0,79)
**Antécédents d'hospitalisation pour accès palustre**				
Oui	41	27	0,007	**2,19(1,23 - 3,9)**
Non	68	98		**0,46(0,26 - 0,82)**
**Age gestationnel au début du TPI**				
Après 28 SA	99	92	0,001	**3,55(1,7 - 7,61)**
Avant 28 SA	10	33		**0,28(0,13- 0,6)**
**Nombre de CPN faites depuis le début de la grossesse**				
〈 4	71(65,1)	86(68,8)	0,554	0,85(0,49 - 1,47)
≥ 4	38(34,9)	39(31,2)		1,18(0,68 - 2,04)
**Nombre de doses de SP**				
〈 3	97	93	0,004	**2,78(1,35- 5,72)**
3	12	32		**0,36(0,17-0,74)**
1 dose	61	51	0,021	**1,84(1,09 – 3,09)**
〉 1	48	74		**0,54(0,32 - 0,91)**

**Tableau 2:** Régression logistique

Variables	Odds Ratio	*IC 95%	P-Value
Primiparité	**2,0122**	**1,1 - 3,7**	**0,0240**
Antécédent d'hospitalisation pour Paludisme	**2,8323**	**1,50 - 5,4**	**0,0014**
Début TPI après 28 semaines	5,3023	0,88 - 32,1	0,0693
Non utilisation de la Milda	1,8160	0,95 - 3,47	0,0714
Moins de 3 doses de SP	1,3892	0,75 - 2,56	0,2920

## Discussion

L'apparition des mutants résistants du plasmodium falciparum à la SP a été décrite à Yaoundé par Chauvin [11] et al en 2015 mais leur importance reste marginale. La faible prévalence de cette résistance ne pourrait à elle seule expliquer l’échec du TPIp-SP. La primiparité a été identifiée comme étant un facteur associé à l’échec du TPI à la SP. La femme enceinte n'est pas plus exposée au paludisme que toute autre personne vivant en zone d'endémie. Mais, la grossesse qui modifie son immunité la rend plus vulnérable. Bien que cette vulnérabilité soit plus élevée pendant la grossesse, Leke [12] et al ont montré que l'immunité vis-à-vis du paludisme augmente avec la parité. Takem [13] et al ont trouvé que la gravidité est un déterminant clé de l'utilisation du TPI. Les multipares utilisent plus volontiers cette prophylaxie que les primipares. Notre étude a trouvé que 54,1% des femmes enceintes sous TPI faisait un accès palustre lorsque le délai entre deux doses de SP dépassait 45 jours. Les données de pharmacovigilance disponibles sur la SP imposent son utilisation seulement après la morphogenèse et un délai de 4 semaines entre deux prises de SP est souhaitable afin de diminuer les effets secondaires chez la mère [14]. Selon les recommandations de l'OMS, le délai entre deux CPN peut dépasser deux mois. MBU [9] et al avaient trouvé que ce délai est souvent supérieur à 10 semaines entre la 1^ère^ et la 2 ^ème^ dose de SP et qu'il semble être responsable du paludisme clinique chez les femmes enceintes sous TPIp-SP, corroborant ainsi nos trouvailles. La prévalence du paludisme est plus élevée au 1^er^ et au 2 ^ème^ trimestres de la grossesse et diminue au 3ème trimestre pour rejoindre le taux avant l'accouchement [15]. C'est ce qui pourrait expliquer pourquoi nous avons trouvé que la prise de la 1^ère^ dose de SP prise après la 28 ^ème^ semaine de grossesse a augmenté le risque d’échec du TPIp-SP. De plus un début tardif de CPN pourrait être incriminé puisque la SP est administré au décours celle-ci.

L’âge gestationnel au moment de l'accès palustre était compris entre 24 à 32 semaines, semblable aux trouvailles de MBU[9] et al et pourrait être en rapport avec un long délai entre la 1^ère^ et la 2 ^ème^ dose de SP. Les femmes ayant fait un accès palustre malgré le TPIp-SP étaient souvent celles qui avaient déjà eu une forme grave ayant nécessité une hospitalisation. La prédisposition individuelle vis-à-vis de la maladie pourrait donc être incriminée. La plupart des femmes enceintes sous TPIp-SP ont eu leur accès palustre au début du 3^ème^ trimestre, période cruciale de la croissance fœtale. Débuter le traitement le plus tôt possible en début de grossesse et respecter le délai entre deux prises sans apparition de la maladie sont les contraintes de cette prophylaxie. L'administration de moins de trois doses de SP s'est révélée être un facteur d’échec du TPIp-SP. De nombreux auteurs ont affirmé que l'administration de trois doses ou plus de SP à la femme enceinte a montré un effet bénéfique plus important pour la mère et l'enfant sans effets délétères graves supplémentaires [16, 17] Depuis 2014, L′OMS recommande que le TPIp-SP soit administré à toutes les femmes enceintes lors de chaque consultation prénatale programmée jusqu′au moment de l′accouchement, à condition que les doses soient administrées à au moins un mois d′intervalle [3]. Kayentao [18] et al ont trouvé que le paludisme placentaire est moins fréquent chez les femmes enceintes ayant reçu trois doses de SP ou plus. Les femmes ayant pris seulement une dose de SP ont un risque plus important d’échec à la prévention. Nous avons trouvé que la non utilisation de la MILDA a contribué à l’échec du TPI. L'utilisation des moustiquaires imprégnées de répulsifs diminue les piqures nocturnes de moustiques, limitant ainsi l'infestation par le plasmodium. Calvin Ebai Bisong [8] et al (2013) ont trouvé que pour de multiples raisons, 17,5% qui possèdent une moustiquaire ne l'utilisent pas en réalité. Selon l'OMS, il convient d'encourager les femmes à utiliser des moustiquaires imprégnées d'insecticides tout au long de leur grossesse car le TPIp-SP ne pourrait se substituer au MILDA [3, 8].

## Conclusion

Les antécédents d'hospitalisation pour paludisme et la primiparité sont des facteurs de risque de l’échec de la prophylaxie anti palustre chez la femme enceinte. Nous recommandons la recherche du paludisme pendant la grossesse malgré la prophylaxie et un renforcement des capacités des prestataires de CPN dans l'application des recommandations de l'OMS pour le TPIp-SP.

### Etat des connaissance sur le sujet

- La chimioprophylaxie est efficace dans la prévention du paludisme en grossesse
- L'association de plusieurs mesures prophylactiques améliore la prévention du paludisme
- Les femmes jeunes célibataires primipares peu éduquées sont plus à risque de paludisme

### Contribution de notre étude a la connaissance

- Les antécédents de paludisme de la patiente sont à considérer dans l'efficacité de la chimioprophylaxie
- Celle-ci doit être précoce pour être plus efficace
- Il faut rechercher et traiter le paludisme en grossesse même en cas de chimioprophylaxie bien conduite
